# Pulse crops: nutrient density, affordability, and environmental impact

**DOI:** 10.3389/fnut.2024.1438369

**Published:** 2024-08-23

**Authors:** Adam Drewnowski, Zach Conrad

**Affiliations:** ^1^Center for Public Health Nutrition, University of Washington, Seattle, WA, United States; ^2^Department of Kinesiology, William & Mary, Williamsburg, VA, United States; ^3^Global Research Institute, William & Mary, Williamsburg, VA, United States

**Keywords:** pulses, protein, national food prices, affordability, greenhouse gas emissions, sustainability, nutrient rich food index

## Abstract

**Introduction:**

Sustainable foods need to be nutrient-rich, affordable, environmentally friendly, and socially acceptable. Pulses, which include beans, lentils, chickpeas, and dried peas are a food group that can fit all those criteria.

**Methods:**

These concepts were tested serially using nutrient profiling methods that focused on protein and were extended to include food prices, greenhouse gas emissions, and energy demand. The present sustainability analyses were based on the US Department of Agriculture (USDA) nutrient composition and food prices data. Environmental impact data came from life cycle assessments (LCA). First, the USDA Protein Foods Group was disaggregated into animal and plant proteins. Plant proteins were separated into pulses, soy products, and nuts and seeds.

**Results:**

Pulses were among the lowest-cost protein sources (per 100 g and per 100 kcal) and had the lowest greenhouse gas emissions GHGE and energy demand. Pulses were among the most sustainable foods when monetary and energy costs were expressed per 50 g of protein (equivalent to 100% DV). Pulses scored well on the Nutrient Rich Food (NRF9.3) nutrient profiling system and on the related Affordable Nutrition Index that assessed nutrient density per penny.

**Discussion:**

Pulses are a source of low-cost plant-based protein and a variety of priority vitamins and minerals, have low carbon footprint and energy demand, and are a valued culinary ingredient across diverse regions and cultures. As dietary guidance turns to plant-based diets, pulses need to be integrated into the global sustainability framework.

## 1 Introduction

Plant-based foods and plant-forward diets offer the promise of improved nutrition, greater affordability, and lower environmental footprint as compared to some existing diets (1–3). Several high-income countries are currently aiming to reduce the consumption of animal foods and so lower the dietary animal protein-to-plant protein ratio (4). The stated goal is to increase the proportion of plant proteins from the current value of approximately 33% of total protein intakes to 50% plant protein (5), 60% plant protein (6), or even beyond (7). Such initiatives are being promoted by researchers (1), consumer groups (8), foundations (9), and by some national and local governments (10, 11). The influential EAT-Lancet planetary health diet, designed for the globe, is built around whole grains, fruits, vegetables, nuts, and legumes and is 64% plant-protein based. The 2,500 kcal/day version restricts beef and pork to 7 g/day each but allows up to 50 g/day of dry beans, lentils, and peas (1).

Pulses, which include dry beans, lentils, peas, and chickpeas are excellent sources of plant-based protein, provide high-quality carbohydrates, are low in saturated fat and contain a variety of priority vitamins and minerals (12–14). The present goal was to assess nutrient density, affordability and carbon footprint of pulses as compared to other protein foods, both animal and plant as listed in the food composition tables (15).

The present sustainability analyses relied on databases developed and maintained by the US Department of Agriculture (USDA) and by other federal agencies (15, 16). Pulses were compared to other protein foods, both animal and plant, across three dimensions of sustainability: nutrient content, price, and environmental impact, the latter assessed using greenhouse gas emissions (GHGE) and cumulative energy demand. Nutrient density of pulses and other protein food sources was assessed using the Nutrient Rich Food (NRF9.3) index, a well-established method to assess nutrient density and the healthfulness of foods (17–19). Affordability metrics used national food prices recently released by the USDA (20). Data on greenhouse gas emissions (GHGE) and energy demand came from dataFIELD (21) and other published LCA data (22, 23).

## 2 Methods

### 2.1 Nutrient content, food prices, and environmental impact databases

The nationally representative National Health and Nutrition Examination Survey (NHANES) has a dietary component known as the What We Eat in America (WWEIA) study. The present analyses used the 2017–18 cycle (NHANES 2017–18). Energy content and nutrient composition of foods reported as consumed by NHANES participants came from the USDA Food and Nutrient Database for Dietary Studies (FNDDS 2017–18) (24). Individual food items in the FNDDS 2017–18 (identified by 8-digit codes) are aggregated by the USDA into food groups, food categories, and food subcategories using WWEIA 1-digit, 2-digit, and 4-digit codes (25).

Included in the USDA WWEIA protein foods group are the categories of red meat (beef, pork, lamb, and game), poultry, seafood, eggs, beans and legumes, and nuts and seeds. The beans and legumes category can be disaggregated into subcategories of pulses and soy products, the latter being mostly processed soy. The pulses subcategory includes beans, peas, chickpeas, lentils, and beans with meat. Beans with meat were evaluated separately. Milk and dairy products fall outside the protein food group, even though they contribute high quality protein to the US diet.

Mean national retail prices for 3,231 food codes in the FNDDS 2017–18 database came from recently released USDA data files (26). The USDA Purchase to Plate Price Tool (PPPT) (20) collects scanner data from retailers and converts retail prices (US$ per product) to unit prices (US$ per 100 g edible portion), adjusting for consumer-level losses and waste. Prices were available for approximately 50% of FNNDS foods that represented 97% of total intake by weight (20).

Data on GHGE and energy demand came from the database on Food Impacts on the Environment for Linking to Diets (dataFIELD), which was developed using a systematic review of LCA studies published between 2005 and 2016 (21). The GHGE and energy demand estimates were averaged across studies and were matched to commodities in the 2010 US Environmental Protection Agency (US EPA) Food Commodity Intake Database (FCID) (16). The FCID provides information on the amount of >500 food components in each food reported as consumed by NHANES participants. The FCID data had been matched with the FNDDS 2017–2018 (23). These data had been used in other studies on the environmental sustainability of alternative dietary patterns in the US (22). The databases were merged using WWEIA 8-digit food identification codes.

### 2.2 Derived variables

#### 2.2.1 Nutrient rich foods index

The Nutrient Rich Food Index (NRF), developed in 2009 (17) is a compensatory nutrient profiling (NP) model that is composed of a positive Nutrient Rich (NR*n*) sub score and a negative nutrients to limit (LIM) sub score. The positive NR*n* sub score is based on a variable number *n* of nutrients to encourage, generally protein, fiber, and a variety of vitamins and minerals. The negative LIM sub score is generally based on the same 3 nutrients to limit: saturated fat, total or added sugar and sodium (17–19). Percent daily values per reference amount are calculated using nutrient standards from the Food and Drug Administration values for a 2,000 kcal/day diet (27). The FDA nutrient standards are shown in Table 1.

**Table 1:** Reference daily values (RDV) for nutrients to encourage and nutrients to limit based on a 2,000 kcal diet.

Nutrient	RDV	Nutrient	RDV
Protein	50 g	Saturated fat	20 g
Fiber	28 g	Total sugar	90 g
Calcium	1,300 mg	Added sugar	50 g
Iron	18 mg	Sodium	2,300 mg
Potassium	3,500 mg		
Magnesium	420 mg		
Vitamin A (retinol activity equivalents)	900 RAE		
Vitamin C	90 mg		
Vitamin D	20 mcg		

The US Food and Drug Administration uses 2,000 calories per day for general nutrition advice.

Dietary reference intakes for potassium were set at 3,500 mg and did not follow the FDA standards. The FDA sets the DV for potassium at 4,700 mg/day; however, based on survey studies, less than 3% of the population consumes that amount (28). In 2019, the National Academies updated the Dietary Reference intakes for potassium that now stand at 3,400 mg for men and 2,600 mg/d for women (29, 30). The current opinion is that potassium between 3,500 and 4,700 mg/day has benefits for lowering blood pressure (28). The European Food Safety Authority (EFSA) recommends at least 3,500 mg/day of potassium (31). Whereas nutrient density calculations conducted in the US tend to use FDA standards, calculation conducted in other countries, especially lower income countries, tend to use standards from Codex Alimentarius or the Food and Agriculture Organization of the United Nations.

The commonly used NRF9.3 version of the NRF family of scores is composed of a NR9 sub score that is based on %DV for 9 nutrients to encourage: protein, fiber, calcium, iron, potassium, magnesium, vitamin A, vitamin C, and vitamin D. Those nutrient choices were guided by the FDA requirement that the use of term healthy on food labeling only applied to foods that provided at least 10% of DV for protein, fiber, calcium, iron, vitamin A and vitamin C (32). The current FDA Nutrition Facts Panel also lists %DV for potassium and vitamin D. Dietary surveys of the US population show that many people consume less than recommended amounts of magnesium (33).

Typically, nutrient content is expressed as percent daily value (%DV) per 100 kcal but can also be expressed per 100 g of food (34). The NR9 sub score is the sum of %DV, with each %DV capped at 100%.

The negative LIM sub score is always composed of Maximum Recommended Values (MRVs) for the same 3 nutrients to limit: saturated fat, total or added sugar, and sodium. The existing FDA regulations for the nutrient content claim “healthy” disqualify foods with excess amounts of saturated fat and sodium. Limiting added sugar is a global health policy. Percent MRV for nutrients to limit can be calculated per 100 kcal or per 100 g. The LIM sub score is the sum of %DV, with each %DV capped at 100%.

The final NRF9.3 score is given as the sum of %DV for nutrients to encourage minus the sum of %MRV for the 3 nutrients to limit. Thus NRF9.3 = NR9-LIM. The NRF score has been validated multiple times with respect to independent measures of a healthy diet.

#### 2.2.2 Affordable nutrient density index

Nutrient profiling systems, such as the Nutrient Rich Foods Index, provide information that can be used to compare the nutrient content of foods, but they do not measure affordability. This is an important metric to capture because affordable nutrient density represents one way to evaluate access to sustainable healthy diets (35). Therefore, we used the Affordable Nutrient Density Index to assess energy content and nutritional value of foods in relation to their cost (36). To be nutrition-relevant, such economic indicators are normally expressed in terms of monetary cost per calorie or per nutrient, as opposed to food weight (35). Food prices data are typically collected and expressed per 100 g of food, edible portion. The present analyses converted food prices to prices per 100 kcal of food. The Affordable Nutrient Density index is a simple ratio of nutrient density per 100 kcal to food prices, also calculated per 100 kcal. Such methods help to identify those foods in the protein foods group that provide most nutrient density per penny (36, 37). An alternative way to calculate protein cost is to estimate the monetary cost of providing a given amount of protein from a given food (38), for example 50 g which is equal to 100% of the daily value.

#### 2.2.3 Environmental cost of pulses

Environmental metrics are typically converted to common units to account for differences in measurement. GHGE are expressed as kg CO_2_ equivalents because different greenhouse gases have very different global warming potential. For example, the global warming potential of nitrous oxide (N_2_O) and methane (CH_4_) is up to 265 and 28 times greater than carbon dioxide (CO_2_), respectively, over a 100-year timescale. Energy demand represents the amount of non-renewable energy used throughout the life cycle of a given food, which includes agricultural production, processing, supply chain, and home preparation. At each stage, different forms of energy are used (electricity, natural gas, gasoline, etc.) that embody very different amounts of energy, so these are converted to MJ.

GHGE values (kg CO_2_ equivalents) and energy demand (MJ) are typically expressed relative to their food weight (i.e., per 100 g). The present calculations of GHGE and energy use focused on the environmental cost associated with providing 50 g of protein, equivalent to 100% of the daily value. For purposes of the present analyses, protein was not corrected for digestibility using the Protein Digestibility Corrected Amino Acid Score (PDCAAS) (39) or the Digestible Indispensable Amino Acid Score (DIAAS) (40).

### 2.3 Plan of analysis

Analyses of nutrient density, affordability, and environmental impact used one-way ANOVAs by WWEIA food subcategory.

## 3 Results

### 3.1 The USDA protein foods group has the highest monetary and environmental cost

**Figure 1:**
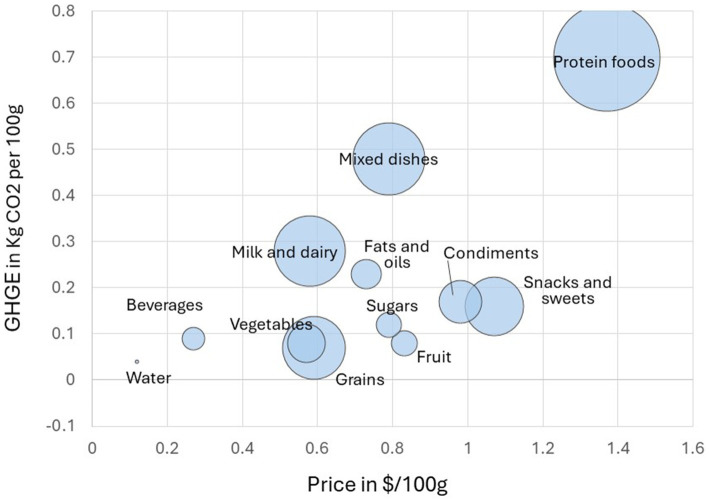
Mean monetary cost ($/100 g) plotted against greenhouse gas emissions (Kg CO_2_eq/100 g) by USDA food group in the FNDDS 2017–18 database. Size of bubble reflects mean protein content in g/100 g for each food group.

The USDA major food groups (identified by WWEIA 1-digit codes) are protein foods, milk and dairy, grains, vegetables, fruits, mixed dishes, snacks and sweets, fats and oils, condiments, and sugars. Figure 1 shows mean national food prices (in $/100 g of food) plotted against the mean carbon footprint (in kg CO_2_-eq/100 g of food) by food group. The size of the bubble reflects the mean protein content of the food group in g/100 g. The USDA protein food group had the highest protein content (>20 g/100 g) and also the highest monetary cost and GHGE. Consistent with past reports, national retail prices per 100 g were highest for the USDA Protein Foods Group (41) and so were greenhouse gas emissions (42). However, those figures generally apply to animal source proteins (42). Subsequent analyses focused on the Protein Foods Group only, and separated animal proteins from plant proteins.

### 3.2 Protein food categories and subcategories

The protein foods group was then disaggregated into categories and subcategories, based on WWEIA codes (25). First, the red meat category was separated into beef, pork, lamb, and cured meats. There was a separate category of organ meats (liver). Poultry was separated into chicken and turkey (43). Plant proteins were separated into beans and legumes, and nuts and seeds. The beans and legumes subcategory was then separated into pulses, pulses with meat, and soy products (mostly processed soy).

**Figure 2:**
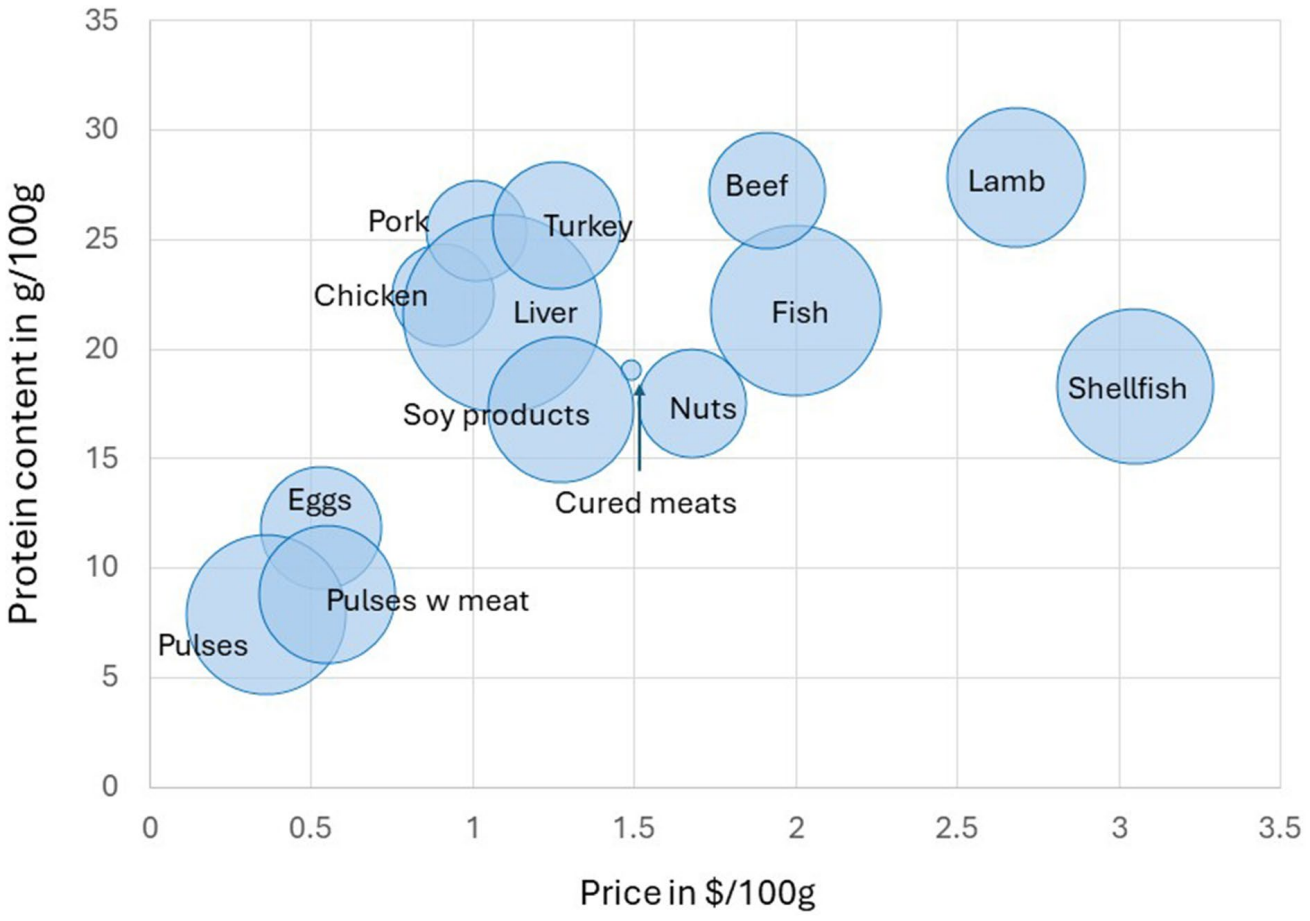
Protein content (g/100 g) plotted against monetary cost ($/100 g) by USDA protein foods subcategory with nutritional profiling. The size of bubble reflects the Nutrient Rich Food Index NRF9.3 value.

Figure 2 shows protein content in g/100 g of food, by food category, plotted against prices ($/100 g). The size of the bubble reflects the relative mean nutrient density of each food category, as indexed by the NRF9.3 score. Most meats and fish had a mean protein content of >20 g per 100 g. Foods that were highest in protein were pork, turkey, beef, and lamb. Pork items contained a mean of 25.4 g of protein per 100 g, as compared to 27.3 g/100 g for beef and 23.2 g/100 g for chicken (43). Pulses on average provided under 10 g of protein per 100 g.

On the other hand, pulses were the lowest cost protein source, with mean prices well below all animal proteins, other than eggs. The highest food prices per 100 g were for shellfish, lamb, fish, and beef. Consistent with the Thrifty Food Plan 2021 report (41), shellfish were much more costly compared to other protein foods. Per 100 g of food, mean national prices for pork were below beef and fish and closer to chicken and turkey (41).

Consistent with past observations, the highest NRF9.3 nutrient density scores (i.e., larger circles represent higher NRF9.3 scores) were observed for liver, fish, pulses, shellfish, and soy products. The lowest values were obtained for cured meats.

### 3.3 Protein content of foods in relation to GHGE and energy demand

**Figure 3:**
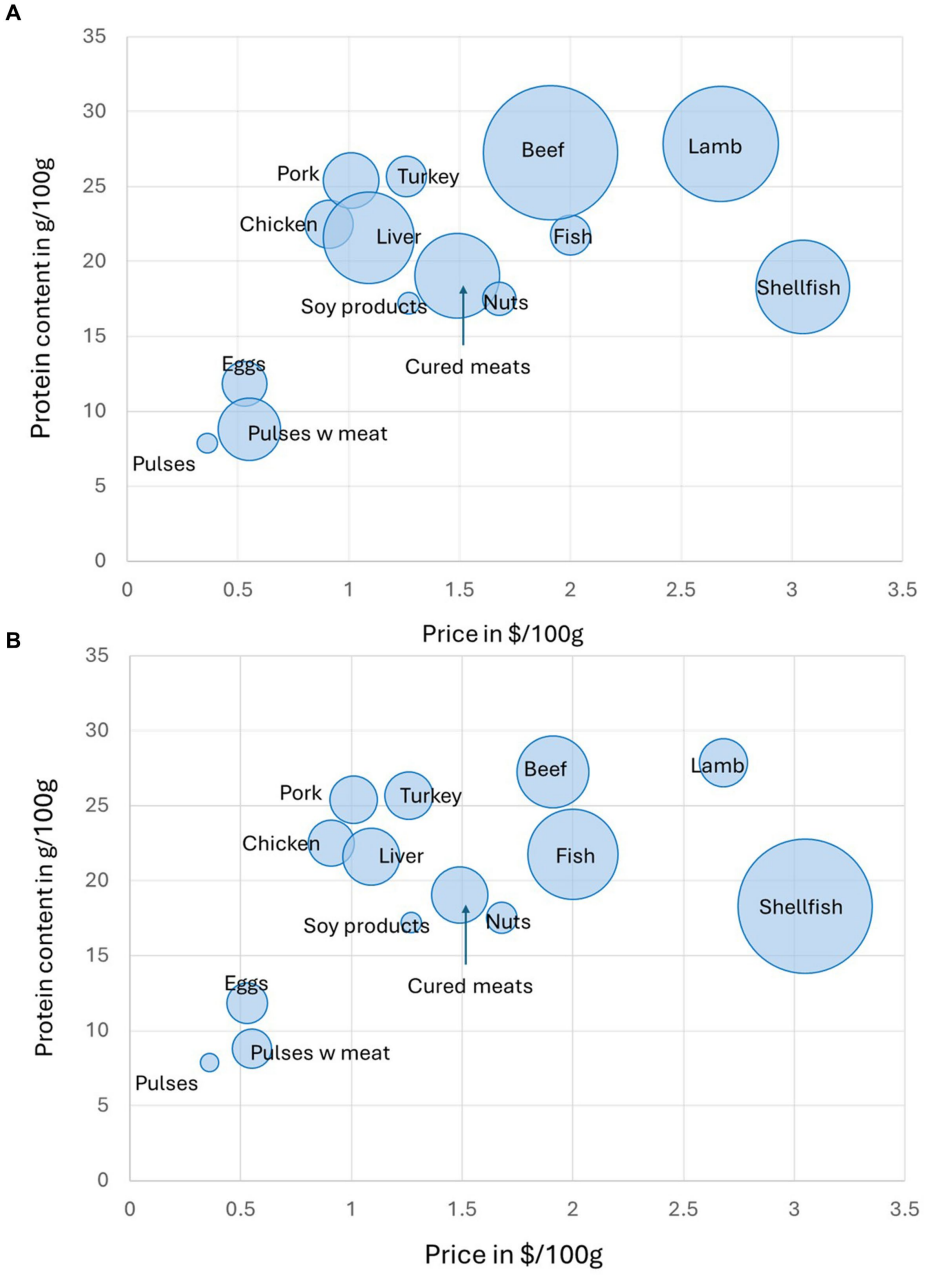
Protein content (g/100 g) plotted against monetary cost ($/100 g) by USDA protein foods subcategory with environmental impacts. **(A)** The size of bubble reflects greenhouse gas emissions (Kg CO_2_eq/100 g) GHGE; **(B)** The size of the bubble reflects energy demand per 100 g.

Figure 3A shows mean food prices ($/100 g) again plotted by mean protein content (g/100 g) by food category, but the size of the bubble is now proportional to the estimated mean GHGE values by category. The highest GHGE values were for beef, lamb, shellfish, liver, and cured meats. Figure 3B also shows mean food prices ($/100 g) plotted against mean protein content (g/100 g) by food category but the size of the bubble is now proportional to the energy demand per 100 g. The highest energy demand was for shellfish and fish followed by cured meats. Compared to meats and fish, pulses had lower protein content. However, in every case, pulses benefited from the lowest prices and the lowest environmental cost.

Mean national food prices and environmental cost by protein food subcategory are also shown in Table 2. Pulses had the lowest monetary cost, GHGE values, and energy demand (all calculated per 100 g).

**Table 2:** Protein food group subcategories and their monetary, greenhouse gas emissions in kg CO_2_ eq per 100 g (GHGE) and energy demand values.

Food category	*N*	Price/100 g	SEM	Food category	*N*	GHGE*/100 g	SEM	Food category	*N*	Energy demand 100 g	SEM
Pulses	50	0.36	0.04	Pulses	64	0.07	0.00	Pulses	64	0.42	0.02
Eggs	102	0.53	0.02	Soy products	20	0.08	0.01	Soy products	20	0.52	0.09
Pulses w meat	5	0.55	0.18	Nuts	78	0.19	0.02	Nuts	78	1.15	0.08
Chicken	146	0.91	0.03	Fish	188	0.28	0.01	Pulses w meat	7	1.90	0.30
Pork	53	1.01	0.03	Turkey	38	0.29	0.01	Eggs	138	2.02	0.05
Liver	11	1.09	0.26	Eggs	138	0.34	0.01	Chicken	169	2.61	0.02
Turkey	26	1.26	0.08	Chicken	169	0.4	0.00	Pork	70	2.74	0.05
Soy products	17	1.27	0.14	Pork	70	0.53	0.01	Turkey	38	2.77	0.10
Cured meats	89	1.49	0.10	Pulses w meat	7	0.67	0.15	Lamb	25	2.85	0.30
Nuts	73	1.68	0.12	Cured meats	110	1.23	0.11	Cured meats	110	3.81	0.16
Beef	42	1.91	0.11	Liver	12	1.44	0.38	Liver	12	3.93	0.62
Fish	139	2.00	0.11	Shellfish	68	1.48	0.15	Beef	59	6.34	0.09
Lamb	29	2.68	0.09	Lamb	25	2.28	0.32	Fish	188	9.89	0.28
Shellfish	45	3.05	0.22	Beef	59	3.05	0.05	Shellfish	68	21.97	0.99

Each set of columns has been arranged from lowest to highest cost. *GHGE, Greenhouse gas emissions.

### 3.4 Affordable nutrient density index

**Figure 4:**
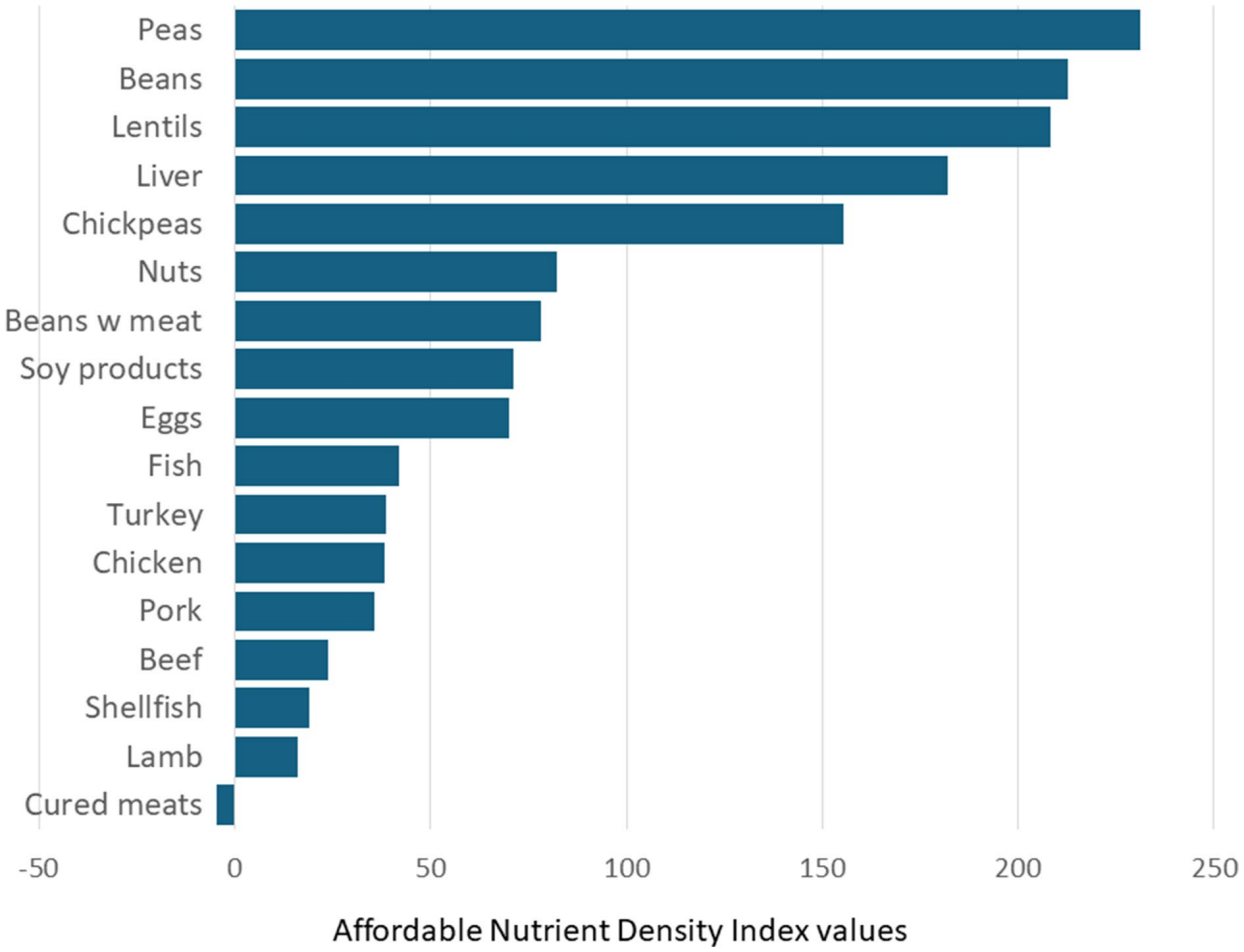
Affordable nutrient rich food index (NRF9.3) values for protein foods by subcategory.

Subsequent calculations focused on nutrient density per unit cost. The affordability index is generally calculated by dividing nutrient density scores calculated per 100 kcal by food prices, also calculated per 100 kcal (36). The metric of affordable nutrient density is shown in Figure 4. Here we divide nutrient density NRF9.3 scores per 100 kcal by food price also per 100 kcal. Consistent with previous reports on the global cost pf priority micronutrients (44, 45), the most affordable nutrient rich foods were pulses (peas, beans, lentils, and chickpeas), organ meats (liver) and nuts.

### 3.5 The monetary and environmental cost of 50 g (100% DV) protein

Table 3 shows the monetary and environmental cost per 50 g of protein by category. Pork meat provided 100% DV of protein for a price that was closer to chicken and eggs than it was to lamb or beef (43). This was the result of the high protein content of pork meat and relatively low price as compared to non-poultry meats (43). Other low-cost protein sources were chicken, eggs, turkey, and pulses. Shellfish were the most expensive protein source, whether per 100 g or per 50 g of protein. The high price of shellfish was also noted in the TFP 2021 report (41).

**Table 3:** Protein food group subcategories and their monetary, greenhouse gas emissions in kg CO_2_ eq per 100 g (GHGE) and energy demand values.

Food category	*N*	Price/50 g Protein	SEM	Food category	*N*	GHGE*/50 g Protein	SEM	Food category	*N*	Energy demand/50 g Protein	SEM
Pork	53	2.00	0.11	Pulses	64	0.44	0.03	Pulses	64	2.92	0.22
Chicken	146	2.16	0.08	Turkey	38	0.55	0.03	Soy products	20	4.28	2.53
Eggs	102	2.25	0.06	Soy products	20	0.57	0.26	Nuts	78	4.29	0.44
Turkey	26	2.42	0.18	Fish	188	0.67	0.02	Lamb	25	5.29	0.57
Pulses	50	2.51	0.34	Nuts	78	0.70	0.06	Turkey	38	5.37	0.21
Liver	11	3.06	0.99	Chicken	169	0.91	0.01	Pork	70	5.76	0.28
Pulses w meat	5	3.27	0.69	Pork	70	1.11	0.05	Chicken	169	5.95	0.07
Beef	42	3.56	0.22	Eggs	138	1.44	0.02	Eggs	138	8.47	0.12
Cured meats	89	3.81	0.20	Liver	12	3.6	1.07	Liver	12	9.83	1.93
Fish	139	4.65	0.22	Cured meats	110	3.65	0.33	Pulses w meat	7	11.35	1.83
Lamb	29	5.05	0.21	Pulses w meat	7	3.83	0.85	Cured meats	110	11.5	0.69
Nuts	73	6.24	0.67	Lamb	25	4.38	0.63	Beef	59	11.94	0.28
Shellfish	45	9.22	0.66	Shellfish	68	4.42	0.45	Fish	188	24.01	0.80
Soy products	17	9.76	4.96	Beef	59	5.85	0.19	Shellfish	68	69.36	3.61

GHGE and energy demand values expressed per 50 g of protein. Each set of columns has been arranged from lowest to highest values. *GHGE, Greenhouse gas emissions.

Pulses had the lowest environmental footprint per 50 g protein when measured in terms of GHGE. Pulses were followed by turkey, soy, and fish. Pulses also had the lowest environmental footprint per 50 g protein when measured in terms of energy demand per 50 g protein. It needs to be mentioned that the present data were not corrected for PDCAAS (Protein Digestibility Corrected Amino Acid Score). Based on still limited PDCAAS data, meats have more digestible protein per 100 g than plant-based proteins, except for soy (39).

## 4 Discussion

The four dimensions of sustainability are nutrition and health, economics, environment and society. Each domain has its own indicators and metrics. To conduct sustainability research, it is necessary to merge siloed data, often maintained by different agencies, to arrive at metrics of nutrient density, affordability, and environmental impact of foods. The lack of data interoperability can be a challenge (46).

The present analyses dealt with three out of four sustainability domains, relying on food composition data and mean national food prices generated by the USDA, as well as life cycle analyses from published studies. The present analyses show that pulses had low cost and low carbon footprint compared to other protein foods and provided low-cost protein (after pork) and the most affordable nutrient density per penny. The present results can be linked to some ongoing food and nutrition policy initiatives in the US and elsewhere.

First, the current edition of DGA 2020–25 recommends that the Healthy U.S.-Style or Healthy Mediterranean-Style dietary pattern ought to include 1.5 cups of pulses per week for adults consuming 2,000 kcal/d (47). The Healthy Vegetarian diet pattern requires another 6 cups per week for a total of 7.5 (47). For comparison, the most recent NHANES data show that adults consume an average 0.12 cups of legumes per day (which includes pulses and soybeans). US adults have a long way to go to meet the recommended intakes for pulses.

As the present analyses show, pulses score high on nutrient density metrics. Among food categories, pulses are unique in providing approximately equal amounts of protein and fiber and are rich in potassium and folate (48). The selection of index nutrients for the present NRF9.3 nutrient profiling model was guided by current federal regulations and Dietary Guidelines for Americans. One advantage of the NRF approach is that is adaptable and can incorporate new nutrients and/or dietary ingredients as new data become available (34).

Modeling studies suggest that replacing protein foods with pulses leads to more nutrient rich and higher quality diets. In one study, a mix of protein foods (meat, chicken, fish, eggs, nuts, seeds, and soy) with the pulses composite in the 2,000 kcal/day US Style Healthy Food Pattern. The substitution od 6–8 oz/week of protein foods with 1.5–2.0 cups of pulses per week increased fiber and decreased cholesterol (49). Higher amounts of pulses generally led to diets with more fiber, iron, magnesium, potassium and copper depending on the type of substitution modeling (49). Another study (50) found that partial replacement of red and processed meats with plant proteins could be done while maintaining sufficient protein and amino acid profiles. The amount of pulses that would provide sufficient nutrients to qualify for nutrient content claims under regional regulatory frameworks was estimated at 100 g or 125 ml (0.5 metric cup) (12).

Second, protein is indeed the costliest nutrient – in terms of both price and environmental cost. However, the USDA protein foods group needs to be disaggregated into animal and plant proteins and those need to be separated into categories. For example, pork has been traditionally put together with red meat in studies on diet quality and health. Once pork was treated as a separate category, it was shown to be distinct in terms of price and environmental cost (43, 51). The present data confirm that pork meat was closer in price to chicken and turkey than to beef and lamb. Meanwhile, pulses can provide consumers with a more sustainable protein option with a high nutritional value per cost (52, 53). That finding may have implications for the redesign of the USDA Thrifty Food Plan, the modeled healthy diet at an affordable cost.

Third, GHGE values and energy demand are typically calculated per weight or volume of food. Arguably, kilogram is not an appropriate measure of nutritional value. There have been attempts to express GHGE values per 1,000 kcal or per 1 kg of protein (54). At least one study suggested that protein ought to be a nutritional functional unit (NFU) in lifecycle analyses (55). An FAO report suggested that a NFU could be based on nutrient density metrics, for example the Nutrient Rich Food Index (NRF) (56).

Fourth, there is explanatory value in disaggregating food groups into their constituent food categories and individual foods. Pulses were further separated into peas, beans, chickpeas, and lentils. The Affordable NRF9.3 gave highest scores to peas, beans, and lentils. These were followed by chickpeas, nuts and beans with meat. When it comes to environmental impact, pulses had the lowest GHGE per 50 g protein – below nuts, fish, soy, and turkey. Calculations of environmental cost of protein were very favorable for pulses.

Finally, there are tradeoffs to be made. Pulses are in the protein group, but their protein content per 100 g is below that of meat and fish (<10 g as opposed to >20 g/100 g). Pulses have a lower protein content than do nuts and soy products (7 g/100 g as opposed to 17 g/100 g for nuts). But pulses compensate for that in that the price per 50 g protein is much lower; effectively pulses offer the most protein (and other key nutrients) per penny.

There are cautions and limitations. The present analyses used combined publicly available USDA and other federal agency databases to evaluate multiple domains of sustainability, consistent with the foundational FAO framework (56) and with past research (57, 58). However, there were data gaps and prices and environmental impact data were not available for all foods in the FNDDS 2017–18 database. For example, the prices do not reflect recent inflation. The Purchase to Plate Price Tools (PPPT) used in this study was derived by linking retail grocery scanner data with the USDA Food and Nutrient Database for Dietary Studies (FNDDS). The USDA-generated PPPT allows users to link data on food intake with price estimates per 100 g edible portion. In 2023 the USDA made Purchase to Price National Average Prices (PP-NAP) available for NHANES cycles 2011–2018. Data for 2019–20 are expected no earlier than fall 2025. Data for 2023–4 are not available at this time. Given the complexities of food inflation, we have made no independent attempt to adjust to post-COVID 2023 or 2024 prices.

Similarly, data on environmental impact of foods were limited to greenhouse gas emissions and energy demand. Energy demand refers to the estimates of energy required for food production (23) Those published data have been derived from multiple sources, both in Europe and the US (59). One problem with the available data on energy, land and water use is that they are rarely context specific. The environmental cost of food products varies by climate and geographic location, For example, pooled data for the classic paper by Poore and Nemecek (54) came from 38,000 farms in 119 countries.

Given the lack of PDCAAS values for FNDDS foods, the data could not be corrected for r protein digestibility. PDCAAS values for pulses are in the order of 0.80 rather than 1.00 (39). This is an important caution and this issue will need to be addressed in future calculations. In practice, pulses have been combined with other foods to meet indispensable amino acid requirements. Future analyses may need to take combinations of foods into account. Further, pulses contain phytates that interfere with iron absorption (60–62). Phytate content can be reduced by soaking and cooking (61).

The present analyses were based on in foods consumed by NHANES participants that included a variety of pulses and pulse dishes. It is important to note that pulses are also used in the manufacture of alternative plant proteins developed from, e.g., pea protein isolates. The present analyses did not examine protein concentrates and isolates that are used to manufacturers plant-based alternatives to milk and meat. Replacing animal protein in the diet may take the form of more plant foods or more alternative proteins (63).

Indicators of the social value of pulses globally were not examined. Pulses are a key component of the Mediterranean diet (64) and are widely recommended (65) and accepted in many cultures as culinary ingredients (66). The Food and Agriculture Organization of the United Nations lists 11 types of pulses, namely dry beans, dry broad beans, dry peas, chickpeas, cow peas, pigeon peas, lentils, Bambara beans, vetches, lupins, and other pulses Future studies will examine trends in pulses consumption in the context of rising incomes and the global nutrition transition.

## 5 Conclusion

A shift toward more environmentally sustainable diet patterns should not compromise diet quality or affordability. By combining multiple publicly available databases on food nutrient content, prices, and environmental impacts, and leveraging several nutrient profiling systems, we show that pulses stand out among plant protein foods. Pulses, which include beans, peas, lentils, and chickpeas, were the lowest cost source of protein and other nutrients and had the lowest environmental impacts.

## Data Availability

Publicly available datasets were analyzed in this study. This data can be found at: https://www.ars.usda.gov/ARSUserFiles/80400530/pdf/fndds/FNDDS_2017_2018_factsheet.pdf (Accessed 14 May, 2024), https://www.ers.usda.gov/data-products/purchase-to-plate/ (Accessed May 14, 2024), and from the following publications: doi:10.1016/j.ajcnut.2023.04.018; doi: 10.3389/fnut.2022.868485; and doi: 10.1186/s12937-020-00629-6.

## References

[1] Willett W Rockström J Loken B Springmann M Lang T Vermeulen S. Food in the Anthropocene: the EAT–lancet commission on healthy diets from sustainable food systems. Lancet. (2019) 393:447–92. doi: 10.1016/S0140-6736(18)31788-4, PMID: 30660336

[2] Pais DF Marques AC Fuinhas JA. The cost of healthier and more sustainable food choices: do plant-based consumers spend more on food? Agric Food Econ. (2022) 10:18. doi: 10.1186/s40100-022-00224-9, PMID: 35909388 PMC9321292

[3] Sexton AE Garnett T Lorimer J. Framing the future of food: the contested promises of alternative proteins. Environ Plan E Nat Space. (2019) 2:47–72. doi: 10.1177/2514848619827009, PMID: 32039343 PMC6989034

[4] Safe Food Advocacy Europe (2020). Actions and policies related to reduction in the consumption of meat in four countries, UK, France, Germany and Switzerland. Available at: https://www.safefoodadvocacy.eu/wp-content/uploads/2023/03/SAFE_LessBetterMeat_WorkDoc_June2020_final.pdf (Accessed May 14, 2024).

[5] Mariotti F. Animal and plant protein sources and Cardiometabolic health. Adv Nutr. (2019) 10:S351–66. doi: 10.1093/advances/nmy110, PMID: 31728490 PMC6855969

[6] de Jong I. (2023). Health council urges Dutch government to push 60:40 plant-to-animal-based ratio diet. Nutrition Insight Available at: https://ni.cnsmedia.com/a/wGYbLjABuLk= (Accessed May 14, 2024).

[7] Gibbs J Cappuccio FP. Plant-based dietary patterns for human and planetary health. Nutrients. (2022) 14:1614. doi: 10.3390/nu14081614, PMID: 35458176 PMC9024616

[8] European Vegetarian Union (2024). The Plant-Based Manifesto. Vienna, Austria: European Vegetarian Union. Available at: https://www.euroveg.eu/wp-content/uploads/2024/02/Plant-Based-Manifesto-full-final-report-11.pdf (Accessed May 14, 2024).

[9] Driscoll M. (2019). More plant-based eating for the planet. Wevelgem, Belgium: alpro Foundation. Available at: https://a.storyblok.com/f/155293/x/18eb71426f/more-plant-based-eating-for-the-planet.pdf (Accessed May 14, 2024).

[10] Ministry of Food, Agriculture, and Fisheries of Denmark (2023). Danish Action Plan for Plant-based Foods. Copenhagen, Denmark: Ministry of Food, Agriculture and Fisheries of Denmark. Available at: https://en.fvm.dk/fileadmin/user_upload/Dokumentation/Danish-Action-Plan-for-Plant-based-Foods.pdf (Accessed May 14, 2024).

[11] Goodett L. (2024). Amsterdam becomes the first EU capital city to endorse the call for a plant based treaty in response to the climate emergency - plant based treaty. Available at: https://plantbasedtreaty.org/amsterdam-endorses-pbt/ (Accessed May 14, 2024).

[12] Marinangeli CPF Curran J Barr SI Slavin J Puri S Swaminathan S. Enhancing nutrition with pulses: defining a recommended serving size for adults. Nutr Rev. (2017) 75:990–1006. doi: 10.1093/nutrit/nux058, PMID: 29202192 PMC5914352

[13] Mitchell DC Marinangeli CPF Pigat S Bompola F Campbell J Pan Y. Pulse intake improves nutrient density among US adult consumers. Nutrients. (2021) 13:2668. doi: 10.3390/nu13082668, PMID: 34444828 PMC8398140

[14] Wallace T Murray R Zelman K. The nutritional value and health benefits of chickpeas and hummus. Nutrients. (2016) 8:766. doi: 10.3390/nu8120766, PMID: 27916819 PMC5188421

[15] U.S. Department of Agriculture, Agricultural Research Service. (2024). FoodData Central. U.S. Department of Agriculture. Available at: https://fdc.nal.usda.gov/ (Accessed May 14, 2024).

[16] U.S. Environmental Protection Agency, University of Maryland. (2000). Food Commodity Intake Database. FoodRisk. Available from: https://fcid.foodrisk.org/ (Accessed May 14, 2024).

[17] Fulgoni VL III Keast DR Drewnowski A. Development and validation of the nutrient-rich foods index: a tool to measure nutritional quality of foods. J Nutr. (2009) 139:1549–54. doi: 10.3945/jn.108.101360, PMID: 19549759

[18] Drewnowski A Fulgoni V. Nutrient density: principles and evaluation tools. Am J Clin Nutr. (2014) 99:1223S–8S. doi: 10.3945/ajcn.113.073395, PMID: 24646818

[19] Drewnowski A. Uses of nutrient profiling to address public health needs: from regulation to reformulation. Proc Nutr Soc. (2017) 76:220–9. doi: 10.1017/S0029665117000416, PMID: 28595659

[20] U.S. Department of Agriculture, Economic Research Service (2023). Purchase to Plate. Economic Research Service. Available at: https://www.ers.usda.gov/data-products/purchase-to-plate/ (Accessed May 14, 2024).

[21] University of Michigan Center for Sustainable Systems (2018). DataFIELD. Available at: https://css.umich.edu/page/datafield (Accessed May 14, 2024).

[22] Conrad Z Drewnowski A Belury MA Love DC. Greenhouse gas emissions, cost, and diet quality of specific diet patterns in the United States. Am J Clin Nutr. (2023) 117:1186–94. doi: 10.1016/j.ajcnut.2023.04.018, PMID: 37075848

[23] Conrad Z Cyril A Kowalski C Jackson E Hendrickx B Lan JJ. Diet sustainability analyses can be improved with updates to the food commodity intake database. Front Nutr. (2022) 9:868485. doi: 10.3389/fnut.2022.868485, PMID: 35832053 PMC9271970

[24] Beltsville Human Nutrition Research Center (2020). Food and Nutrient Database for Dietary Studies 2017–2018. Beltsville, MD: U.S. Department of Agriculture, Agricultural Research Service. Available at: https://www.ars.usda.gov/ARSUserFiles/80400530/pdf/fndds/FNDDS_2017_2018_factsheet.pdf (Accessed May 14, 2024).

[25] Beltsville Human Nutrition Research Center (2022). What We Eat in America Food Categories for use with WWEIA, NHANES 2017 March 2020 Prepandemic. Beltsville, MD: U.S. Department of Agriculture, Agricultural Research Service. Available at: https://www.ars.usda.gov/ARSUserFiles/80400530/pdf/1720/Food_Category_List_2017-March%202020.pdf (Accessed May 14, 2024).

[26] Carlson AC Tornow CE Page ET Brown McFadden A Palmer ZT. Development of the purchase to plate crosswalk and Price tool: estimating prices for the National Health and Nutrition Examination Survey (NHANES) foods and measuring the healthfulness of retail food purchases. J Food Compos Anal. (2022) 106:104344. doi: 10.1016/j.jfca.2021.104344

[27] U.S. Food and Drug Administration (2024). Daily Value on the Nutrition and Supplement Facts Labels. U.S. Food and Drug Administration. Available at: https://www.fda.gov/food/nutrition-facts-label/daily-value-nutrition-and-supplement-facts-labels (Accessed May 14, 2024).

[28] Weaver CM Stone MS Lobene AJ Cladis DP Hodges JK. What is the evidence base for a potassium requirement? Nutr Today. (2018) 53:184–95. doi: 10.1097/NT.0000000000000298, PMID: 30369637 PMC6181280

[29] Oria M Harrison M Stallings VA. (2019). Dietary reference intakes for sodium and potassium. Washington (DC): National Academies Press (US). Available at: https://www.ncbi.nlm.nih.gov/books/NBK538102/ (Accessed July 27, 2024). 30844154

[30] National Institutes of Health, Office of Dietary Supplements (2022). Potassium-Fact Sheet for Health Professionals. National Institutes of Health. Available at: https://ods.od.nih.gov/factsheets/Potassium-HealthProfessional/ (Accessed May 14, 2024).

[31] European Food Safety Authority Journal (2016). Dietary Reference Values for Potassium. doi:10.2903/j.efsa.2016.xxxx. Available at: https://www.efsa.europa.eu/sites/default/files/consultation/160713.pdf (Accessed July 27, 2024).

[32] U.S. Food and Drug Administration (2024). Use of the term Healthy on food labeling. Available at: https://www.fda.gov/food/food-labeling-nutrition/use-term-healthy-food-labeling (Accessed July 27, 2024).

[33] National Institutes of Health (2022). Magnesium. Available at: https://ods.od.nih.gov/factsheets/Magnesium-HealthProfessional/ (Accessed July 27, 2024).

[34] Drewnowski A. A novel nutrient rich food (NRFa11.3) score uses flavonoids and carotenoids to identify antioxidant-rich spices, herbs, vegetables, and fruit. Front Nutr. (2024) 11:1386328. doi: 10.3389/fnut.2024.1386328, PMID: 38699550 PMC11063353

[35] Drewnowski A Darmon N Monsivais P. Affordable nutrient density: toward economic indicators of sustainable healthy diets. Sustain For. (2021) 13:9300. doi: 10.3390/su13169300

[36] Drewnowski A Rehm CD. Vegetable cost metrics show that potatoes and beans provide most nutrients per penny. PLoS One. (2013) 8:e63277. doi: 10.1371/journal.pone.0063277, PMID: 23691007 PMC3654977

[37] Hess JM Cifelli CJ Agarwal S Fulgoni VL. Comparing the cost of essential nutrients from different food sources in the American diet using NHANES 2011–2014. Nutr J. (2019) 18:68. doi: 10.1186/s12937-019-0496-5, PMID: 31706353 PMC6842517

[38] Drewnowski A Tang W Brazeilles R. Calcium requirements from dairy foods in France can be met at low energy and monetary cost. Br J Nutr. (2015) 114:1920–8. doi: 10.1017/S0007114515003669, PMID: 26450475

[39] Schaafsma G. The protein digestibility–corrected amino acid score. J Nutr. (2000) 130:1865S–7S. doi: 10.1093/jn/130.7.1865S 10867064

[40] Wolfe RR Rutherfurd SM Kim IY Moughan PJ. Protein quality as determined by the digestible indispensable amino acid score: evaluation of factors underlying the calculation. Nutr Rev. (2016) 74:584–99. doi: 10.1093/nutrit/nuw022, PMID: 27452871 PMC6322793

[41] U.S. Department of Agriculture. (2021). Thrifty Food Plan, 2021. Washington (DC): U.S. Department of Agriculture, Food and Nutrition Service. Available at: https://fns-prod.azureedge.us/sites/default/files/resource-files/TFP2021.pdf (Accessed May 14, 2024).

[42] Gaillac R Marbach S. The carbon footprint of meat and dairy proteins: a practical perspective to guide low carbon footprint dietary choices. J Clean Prod. (2021) 321:128766. doi: 10.1016/j.jclepro.2021.128766

[43] Drewnowski A. Perspective: the place of pork meat in sustainable healthy diets. Adv Nutr. (2024) 15:100213. doi: 10.1016/j.advnut.2024.100213, PMID: 38508316 PMC11035016

[44] Beal T Gardner CD Herrero M Iannotti LL Merbold L Nordhagen S. Friend or foe? The role of animal-source foods in healthy and environmentally sustainable diets. J Nutr. (2023) 153:409–25. doi: 10.1016/j.tjnut.2022.10.016 36894234

[45] Beal T Ortenzi F. Priority micronutrient density in foods. Front Nutr. (2022) 9:806566. doi: 10.3389/fnut.2022.806566, PMID: 35321287 PMC8936507

[46] Jennings-Dobbs EM Forester SM Drewnowski A. Visualizing data interoperability for food systems sustainability research-from spider webs to neural networks. Curr Dev Nutr. (2023) 7:102006. doi: 10.1016/j.cdnut.2023.102006, PMID: 37915997 PMC10616130

[47] U.S. Department of Agriculture, U.S. Department of Health and Human Services (2020). Dietary Guidelines for Americans 2020–2025. Available at: https://www.dietaryguidelines.gov/sites/default/files/2020-12/Dietary_Guidelines_for_Americans_2020-2025.pdf (Accessed February 12, 2024).

[48] Didinger C Thompson HJ. Defining nutritional and functional niches of legumes: a call for clarity to distinguish a future role for pulses in the dietary guidelines for Americans. Nutrients. (2021) 13:1100. doi: 10.3390/nu13041100, PMID: 33801714 PMC8066616

[49] Agarwal S Fulgoni VL. Effect of adding pulses to replace protein foods and refined grains in healthy dietary patterns. Nutrients. (2023) 15:4355. doi: 10.3390/nu15204355, PMID: 37892430 PMC10610119

[50] Simojoki M Männistö S Tapanainen H Maukonen M Valsta LM Itkonen ST. The impacts of partial replacement of red and processed meat with legumes or cereals on protein and amino acid intakes: a modelling study in the Finnish adult population. Ann Med. (2023) 55:2281661. doi: 10.1080/07853890.2023.2281661, PMID: 37976343 PMC10732208

[51] Poinsot R Maillot M Drewnowski A. Fresh pork as protein source in the USDA thrifty food plan 2021: a modeling analysis of lowest-cost healthy diets. Nutrients. (2023) 15:1897. doi: 10.3390/nu15081897, PMID: 37111116 PMC10146423

[52] Young L Mackay S Bradbury KE. Nutrient content and cost of canned and dried legumes and plant-based meat analogues available in New Zealand supermarkets. Nutr Diet. (2023) 80:472–83. doi: 10.1111/1747-0080.12834, PMID: 37545013

[53] Madlala SS Hill J Kunneke E Faber M. Nutrient density and cost of commonly consumed foods: a south African perspective. J Nutr Sci. (2023) 12:e10. doi: 10.1017/jns.2022.119, PMID: 36721720 PMC9879879

[54] Poore J Nemecek T. Reducing food’s environmental impacts through producers and consumers. Science. (2018) 360:987–92. doi: 10.1126/science.aaq0216 29853680

[55] McAuliffe GA Takahashi T Beal T Huppertz T Leroy F Buttriss J. Protein quality as a complementary functional unit in life cycle assessment (LCA). Int J Life Cycle Assess. (2023) 28:146–55. doi: 10.1007/s11367-022-02123-z 36685326 PMC9845161

[56] McLaren S Berardy A Henderson A Holden N Huppertz T Jolliet O. (2021). Integration of environment and nutrition in life cycle assessment of food items: Opportunities and challenges. Rome, Italy: Food and Agriculture Organization. Available at: http://www.fao.org/documents/card/en/c/cb8054en (Accessed May 14, 2024).

[57] Drewnowski A. The ecosystem inception team. The Chicago consensus on sustainable food systems science. Front Nutr. (2018) 4:74. doi: 10.3389/fnut.2017.00074, PMID: 29744333 PMC5930345

[58] Drewnowski A Finley J Hess JM Ingram J Miller G Peters C. Toward healthy diets from sustainable food systems. Curr Dev Nutr. (2020) 4:nzaa083. doi: 10.1093/cdn/nzaa083, PMID: 32551411 PMC7288378

[59] Heller MC Willits-Smith A Meyer R Keoleian GA Rose D. Greenhouse gas emissions and energy use associated with production of individual self-selected US diets. Environ Res Lett. (2018) 13:044004. doi: 10.1088/1748-9326/aab0ac, PMID: 29853988 PMC5964346

[60] Petroski W Minich DM. Is there such a thing as “anti-nutrients”? A narrative review of perceived problematic plant compounds. Nutrients. (2020) 12:2929. doi: 10.3390/nu12102929 32987890 PMC7600777

[61] Kumar Y Basu S Goswami D Devi M Shivhare US Vishwakarma RK. Anti-nutritional compounds in pulses: implications and alleviation methods. Legume Sci. (2022) 4:e111. doi: 10.1002/leg3.111

[62] Sinkovič L Pipan B Šibul F Nemeš I Tepić Horecki A Meglič V. Nutrients, Phytic acid and bioactive compounds in marketable pulses. Plants Basel Switz. (2022) 12:170. doi: 10.3390/plants12010170, PMID: 36616298 PMC9824021

[63] Drewnowski A. Perspective: alternative proteins in low-and middle-income countries (LMIC) face a questionable future: will technology negate Bennett’s law? Curr Dev Nutr. (2023) 8:101994. doi: 10.1016/j.cdnut.2023.101994, PMID: 38476727 PMC10926128

[64] Drewnowski A Eichelsdoerfer P. The Mediterranean diet: does it have to cost more? Public Health Nutr. (2009) 12:1621–8. doi: 10.1017/S1368980009990462, PMID: 19689831 PMC2849996

[65] Hughes J Pearson E Grafenauer S. Legumes—a comprehensive exploration of global food-based dietary guidelines and consumption. Nutrients. (2022) 14:3080. doi: 10.3390/nu14153080 35956258 PMC9370574

[66] Winham D Tisue M Palmer S Cichy K Shelley M. Dry bean preferences and attitudes among Midwest Hispanic and non-Hispanic white women. Nutrients. (2019) 11:178. doi: 10.3390/nu11010178, PMID: 30650616 PMC6356900

